# Effects of prenatal and postnatal depression, and maternal stroking, at the glucocorticoid receptor gene

**DOI:** 10.1038/tp.2014.140

**Published:** 2015-05-05

**Authors:** C Murgatroyd, J P Quinn, H M Sharp, A Pickles, J Hill

**Affiliations:** 1 School of Healthcare Science, Manchester Metropolitan University, Manchester, UK; 2 Department of Molecular and Clinical Pharmacology, Institute of Translational Medicine, University of Liverpool, Liverpool, UK; 3 Institute of Psychology, Health and Society, University of Liverpool, Liverpool, UK; 4 Institute of Psychiatry, King's College London, London, UK; 5 School for Psychology and Clinical Language Sciences, University of Reading, Reading, UK

## Abstract

In animal models, prenatal and postnatal stress is associated with elevated hypothalamic–pituitary axis (HPA) reactivity mediated via altered glucocorticoid receptor (GR) gene expression. Postnatal tactile stimulation is associated with reduced HPA reactivity mediated via increased GR gene expression. In this first study in humans to examine the joint effects of prenatal and postnatal environmental exposures, we report that GR gene (*NR3C1*) 1-F promoter methylation in infants is elevated in the presence of increased maternal postnatal depression following low prenatal depression, and that this effect is reversed by self-reported stroking of the infants by their mothers over the first weeks of life.

## Introduction

In animal models, prenatal and postnatal stress cause long-term elevations in hypothalamic–pituitary axis (HPA) reactivity and anxiety-like behaviors. These effects are mediated via altered glucocorticoid receptor (GR) gene expression.^1^ In rodents, maternal licking and grooming over the first days of life cause reduced HPA-axis reactivity and anxiety-like behaviors mediated via increased GR expression accounted for, at least in part, by demethylation at exon 1–7 promoter of the rat GR gene (*Nr3c1*) in the hippocampus of the offspring. These epigenetic changes emerge over the first week of life and persist into adulthood.^2^ Epigenetic modifications are thought to link early-life stress to later susceptibility to behavioral disorders through interference with the development and functioning of the HPA-axis early in life.^3^ The epigenetic process of DNA methylation involves the addition of methyl groups to CpG dinucleotides in gene regulatory regions that associate with repression of gene expression. Translation into humans would have far-reaching consequences for our understanding of the role of early environmental stressors, with implications for health and social policy. Findings consistent with fetal programming of HPA-axis regulation have been reported in humans. Maternal anxiety and depression during pregnancy predict childhood behavior problems after controlling for postnatal environmental exposure,^4, 5^ and prenatal maternal anxiety predicts persistence of behavior problems from childhood to adolescence.^6^ Prenatal maternal depression predicts infant temperamental negative emotionality^7^ and maternal cortisol during pregnancy predicts infant cortisol reactivity to a stressor.^8^

**Figure 1:**
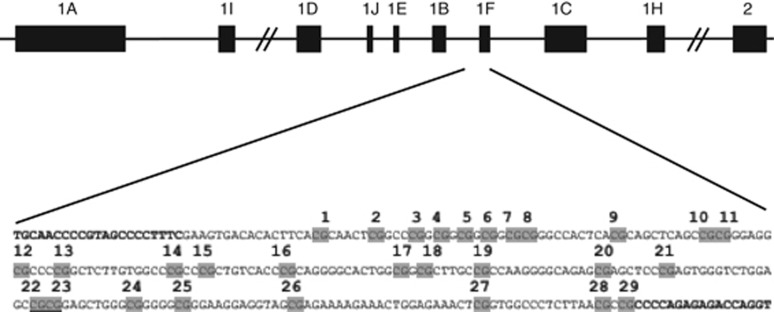
Scheme of the human *NR3C1* gene analyzed by bisulfite pyrosequencing. The 5′-end of the human *NR3C1* gene contains multiple first exons, with multiple transcriptional start sites and mRNA splice variants. The region analyzed by bisulfite pyrosequencing (primer sequences are in bold) contains 29 CpGs (CpG unit 22 and 23 are underlined) and encompasses exon 1-F, which is the human homolog of the rat exon 1–7, previously shown to be differentially methylated.^2^

Animal findings of the epigenetic effects of early-life stress have been validated in humans in a study reporting elevated *NR3C1* 1-F promoter methylation and reduced GR expression in postmortem hippocampal tissue of suicide completers who were abused during childhood, when compared with non-abused.^9^ Other studies using peripheral DNA, from blood or saliva of infants and adolescents, have shown increased levels of *NR3C1* methylation in response to perinatal stress^10, 11, 12^ and abuse or neglect during childhood.^13, 14^ Many further report enduring DNA methylation changes in adulthood following stress or traumatic events such as abuse or neglect in childhood.^9, 13, 14, 15^ Several clinical studies examining leukocytes have reported elevated methylation of the homologous human *NR3C1* 1-F promoter (homologous to the rat 1–7 promoter) at a specific CpG (CpG unit 22,23, Figure 1) associated with prenatal maternal depression^10, 16, 17^ and childhood stress.^14, 18^

Effects of postnatal maternal behaviors reported in animal models have not so far been translated into humans. The postnatal maternal licking and grooming effects on rodent GR expression, *Nr3c1* 1–7 promoter region demethylation,^19^ improved HPA-axis regulation and reduced anxiety behaviors.^20, 21^ are caused by tactile stimulation. We therefore asked whether, in humans, maternal stroking has the effect that would be predicted from the animal work, that is, does it reverse prenatal stress effects? Using a self-report measure on two occasions, we asked mothers participating in the longitudinal Wirral Child Health and Development Study, how often they stroked their infants when they were 5 and 9 weeks old. We found that associations of prenatal depression with vagal reactivity and temperament at 29 weeks of age were both modified by maternal stroking over the first weeks of life.^22^ The significant statistical interaction was that increasing prenatal depression was associated with decreasing vagal reactivity, which is likely to be associated later in life with poorer emotion regulation, only in the infants of low-stroking mothers. Similarly the association between prenatal depression and increasing negative emotionality, as reported by mothers in a standard measure of temperament, was also seen only in the infants of low-stroking mothers. Reporting from the same sample, we have recently shown that maternal stroking interacts with prenatal anxiety to predict child emotional problems at 2.5 years—the association between maternal anxiety and child emotional problems was evident only in the children of low-stroking mothers.^23^ These are the first findings in humans of an enduring effect of maternal stroking on the basis of predictions from animal models. No previous studies have investigated whether *NR3C1* methylation associated with maternal depression is modified by maternal stroking.

In the case of maternal depression, prenatal and postnatal levels are highly correlated, and each has to be accounted for in predicting DNA methylation. Strikingly, animal studies have not yet attempted to mimic the human condition by examining the joint effects of pre- and postnatal stress, and so there is no firm basis from which to predict in humans. We, therefore, examined whether each of pre- or postnatal depression have effects on infant *NR3C1* 1-F promoter DNA methylation at CpG unit 22 and 23, or that they interact to give distinct outcomes. We also investigated whether effects of maternal depression are reversed by maternal stroking.

## Materials and methods

### Design

The participants were members of the Wirral Child Health and Development Study, a prospective epidemiological longitudinal study of prenatal and infancy origins of conduct disorders. This uses a two stage stratified design in which a larger general population sample of first-time mothers was recruited in pregnancy (extensive sample) and from which a subsample was drawn for more intensive assessment (intensive sample). All families in the extensive sample follow a brief assessment protocol while those in the intensive subsample receive more time-consuming detailed assessments such as the observations of mother-infant interactions described in this paper. The design allows general population estimates of means and associations to be derived for all extensive or intensive sample measures.

Approval for the procedures was obtained from the Cheshire North and West Research Ethics Committee (UK). The extensive sample was identified from consecutive first-time mothers who booked for antenatal care at 12 weeks gestation between 12/02/2007 and 29/10/2008. The booking clinic was administered by the Wirral University Teaching Hospital which is the sole provider of universal prenatal care on the Wirral Peninsula. Socioeconomic conditions on the Wirral range between the deprived inner city and affluent suburbs, but with few from ethnic minorities. The study was introduced to the women by clinic midwives who asked for their agreement to be approached by study research midwives when they attended for ultrasound scanning at 20 weeks gestation. After complete description of the study to the women, written informed consent was obtained by the study midwives, who then administered questionnaires and an interview in the clinic.

### Participants

Of those approached by study midwives, 68.4% gave consent and completed the measures, yielding an extensive sample of 1233 mothers with surviving singleton babies. The sampling flow chart has been published previously.^22^ The mean age at recruitment of extensive sample participants was 26.8 years (s.d.5.8, range 18–51). Using the UK Index of Multiple Deprivation (IMD)^24^ based on data collected from the UK Census in 2001, 41.8% of the extensive sample reported socioeconomic profiles found in the most deprived UK quintile, consistent with the high levels of deprivation in some parts of the Wirral. Forty eight women (3.9%) described themselves as other than white British. Demographic and antenatal stratification measures were administered at 20 weeks gestation with all extensive sample participants.

A stratified random subsample of 316 mothers was recruited to the intensive sample at 32 weeks gestation with the sampling fraction depending on their prior responses to a measure of partner psychological abuse on entry into the extensive study at 20 weeks gestation.^22^ In addition to assessments of the mothers at 20 and 32 weeks gestation, mothers and infants generated data at 5, 9, and 29 weeks, and at 14 months. Two hundred and sixty eight mothers and infants came into the lab at 14 months for detailed observational, interview and physiological measures. Seven parents declined consent for DNA collection, 3 samples were spoilt, and 25 assessments were curtailed before saliva collection because of time constraints. Sufficient DNA for methylation analyses was obtained from 181 infants.

### Measures

#### Maternal depression

Maternal symptoms of depression were assessed at 20 and 32 weeks' gestation, and when infants were 5, 9 and 29 weeks, and 14 months, using the Edinburgh Postnatal Depression Scale (EPDS) which has been used extensively to assess pre- and postnatal depression.^25^ The measure was designed specifically to avoid confounding by symptoms commonly experienced by non-depressed women shortly after childbirth.

#### Maternal stroking

Maternal stroking was assessed by self report using The Parent–Infant Caregiving Scale (Sharp *et al.*, 2012) in which mothers completed four items reporting on how often (1=never, 2=rarely, 3=sometimes, 4=often, 5=a lot) they currently stroked their baby's face, back, tummy, arms and legs. The four stroking items assess a stroking construct as evidenced in high loadings of all of the items on a latent variable^22^ and test–retest reliability over 4 weeks is acceptable (*r*=0.58). Separate analyses were conducted with stroking at 5 and 9 weeks.

#### DNA methylation

Methylation status in the *NR3C1* 1-F promoter was examined at the same CpGs (CpG unit 22 and 23, see Figure 1) identified by Oberlander *et al.,*^10^ Conradt *et al.,*^16^ Hompes *et al.,*^17^ Tyrka *et al.*^14^ and Melas *et al.*^18^ DNA collected from Oragene saliva samples was extracted, bisulphite treated, amplified (Forward, 5′-GACCTGGTCTCTCTGGGG-3′; Reverse, 5′-TGCAACCCCGTAGCCCCTTTC-3′) and run on a Sequenom EpiTYPER system (Sequenom, San Diego, CA, USA). Data were transformed to percentage of methylation at CpG unit 22 and 23 to allow for comparison with previous analysis of differential methylation at this locus.

#### Stratification variable and confounders

Partner psychological abuse was assessed using a 20-item questionnaire covering humiliating, demeaning or threatening utterances in the partner relationship during pregnancy over the previous year.^26^ Strata were defined using the highest of the partner-to-participant and participant-to-partner scores for each family. The sampling fraction for participation in the intensive sample was higher in the high-risk stratum than the low-risk stratum and, as described in the analysis section, stratum weights were used to account for this selection on our results.

Maternal age (at this first pregnancy) and marital status at 20 weeks' gestation were included because of their associations with maternal depression in this sample (Sharp *et al.*, in press). Socioeconomic status was included because of its established association with adult depression.^27^ Socioeconomic status was determined using the revised English Index of Multiple Deprivation (IMD)^24^ on the basis of the data collected from the UK Census in 2001. According to this system, postcode areas in England are ranked from most deprived (that is, IMD of 1) to least deprived (that is, IMD of 32 482) on the basis of neighborhood deprivation in seven domains: income, employment, health, education and training, barriers to housing and services, living environment and crime. All mothers were given IMD ranks according to the postcode of the area where they lived and assigned to a quintile on the basis of the UK distribution of deprivation. Information about drinking alcohol and smoking was obtained at 20 and 32 weeks' gestation and was included because of published associations with altered DNA methylation.^28, 29^ Sex differences in the DNA methylation patterns have been reported,^29^ and birth records were used to determine the sex of the infant. Birth weight was also obtained from birth records and birth weight by gestational age was used as a measure of fetal growth. Low fetal growth is associated with elevated fetal glucocorticoid exposure and so might be associated with elevated GR gene methylation.^29^ Obstetric risk was rated using a weighted severity scale developed by a collaboration of American and Danish obstetricians and pediatric neurologists.^30, 31^ The scale has 32 items, each of which has an assigned score in the range 1–5, and the highest rated item provides the value for analyses. It has been used widely in studies of perinatal complications and later development.

### Statistical analysis

All analyses were undertaken in Stata 13 (StataCorp, 2012). The two-phase stratified sample design allows estimates to be reported for the general population from the stratified subsample by the use of inverse probability weights. Weights took account not only of the original stratification but also of the sample attrition that took place up to the assessment and methylation assay at age 14 months including mothers' age and years of education, maternal smoking and depression score in pregnancy, and a score of the number of items left incomplete at the initial assessment. To avoid undue influence of some extreme observations of rates of methylation, the rates were grouped into seven categories of methylation level with approximately equal frequency (septiles) and association with other variables analyzed by means of weighted ordinal logistic regression. Reported effect estimates are thus log-odds coefficients. Stata's svy option was used with standard errors and *P*-values based on the robust estimator of the parameter covariance matrix. Variation in the weights associated with the covariates of each model was removed to improve efficiency. Predictions of methylation levels were examined first including only variables of interest, and then after adding potential confounders for obstetric risk index, self-reported maternal smoking at 20 and 32 weeks of pregnancy, self-reported alcohol consumption at 20 weeks, birth weight by gestational age, neighborhood deprivation, maternal age, marital status and 20-week psychological abuse score.

**Figure 2:**
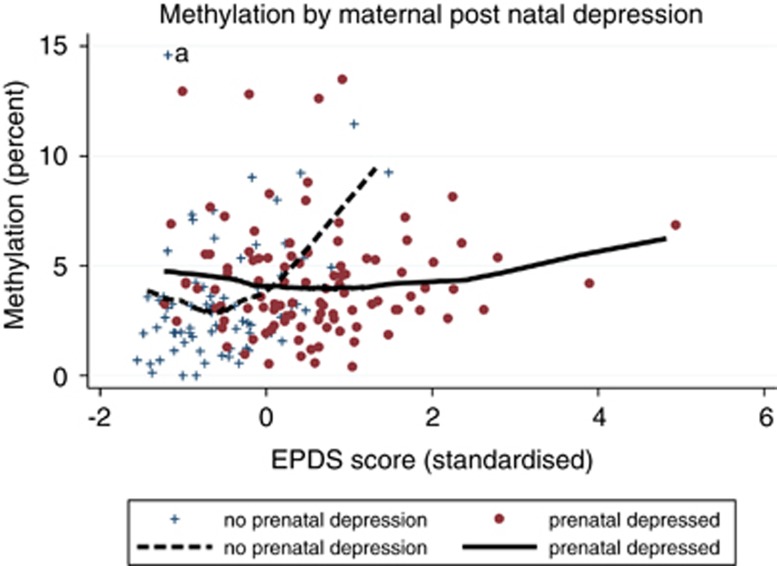
Child *NR3C1* 1-F promoter methylation percent by standardized maternal postnatal depression scores. The figure gives the locally weighted scatterplot smoothing (LOWESS) plots showing how the child's raw methylation percent increases with increased maternal postnatal depression for those with low maternal prenatal depression (dashed line) but not those with high prenatal depression (solid). To improve visualization, the point marked ‘a' has been displaced (from methylation 29%) in the scatterplot (but conservatively retained in the LOWESS).

**Figure 3:**
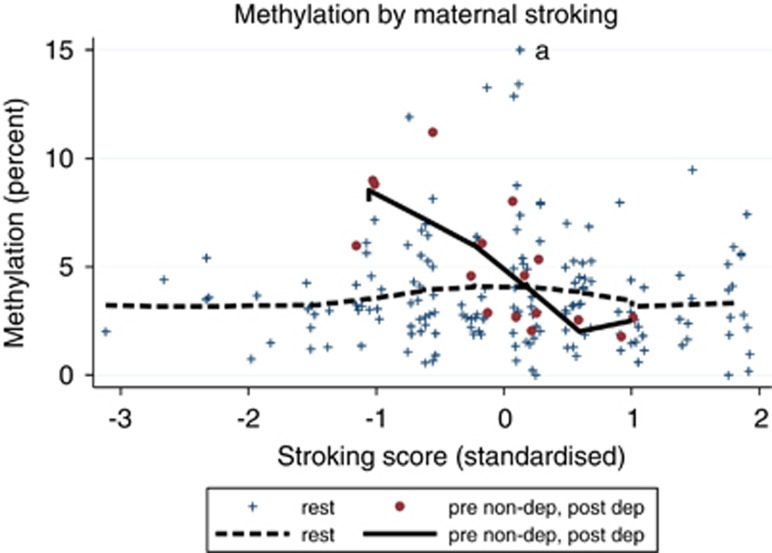
Child *NR3C1* 1-F promoter methylation percent by standardized maternal stroking scores. The figure gives the locally weighted scatterplot smoothing plots showing how the child's raw methylation percent decreases with maternal stroking for children with mothers who reported low prenatal but high postnatal depression scores (solid line). No such decrease is seen for the remainder of the children (dashed).

Figures 2 and 3 show locally weighted scatterplot smoothing plots^32^ fitted to the raw methylation data. These are not based on model-predicted values but are empirical plots and are unweighted. The locally weighted scatterplot smoothing plots were fitted to the original raw data; whereas for the scatter plots, one marked observed methylation value was recoded from 29 to 14 to improve visualization.

## Results

Maternal depression (EPDS) scores at 20 weeks' gestation were strongly associated with mean EPDS across the four postnatal assessment points (*r*=0.68). In separate ordinal logistic regression analyses, elevated methylation in the infants were predicted by EPDS scores at 20 weeks of pregnancy (log-odds coefficient=0.348, s.e.=0.139, *P*=0.013) and mean postnatal EPDS scores (coefficient=0.574, s.e.=0.141, *P*<0.001). When examined jointly, the interaction between 20 weeks' prenatal and mean postnatal depression scores was significant (coefficient=−0.418, s.e.=0.207, *P*=0.045). The effect of the interaction on raw methylation percentage in the infants is illustrated in Figure 2, where groups below and above the median 20 weeks' EPDS scores are contrasted. It can be seen that increasing postnatal depression was associated with increasing methylation only in infants from mothers below the median for prenatal depression.

We hypothesized that if maternal stroking reverses the effects of prenatal and postnatal depression on *NR3C1* 1-F promoter methylation, it should be associated with reduced methylation in the children of mothers with the combination of low prenatal and high postnatal depression. In view of the evidence that in rodents the effect of licking and grooming is limited to a short postnatal critical period, the effects of stroking at 5 and at 9 weeks were analyzed separately. Because low maternal prenatal depression is associated with low postnatal depression, the group that we identified below the median on prenatal depression and above the median on postnatal depression was relatively small (*N*=16). These children had substantially higher methylation levels than the other 165 (coefficient=1.688, s.e.=0.510, *P*=0.001). Increased maternal stroking at 5 weeks specifically reduced methylation in this group as evidenced in a highly significant statistical interaction between the membership of this group and maternal stroking when infants were 5 weeks old (coefficient=−2.754, s.d.=0.573, *P*<0.001). This interaction was unaltered after the addition of confounders to the model (coefficient=−2.634, s.e.=0.567, *P*<0.001). The interaction is illustrated in Figure 3, where it can be seen that with increasing maternal stroking, *NR3C1* 1-F promoter methylation in the children of mothers in the low prenatal and high postnatal group fell to the level of the remainder of the sample. By contrast, there was no effect of maternal stroking at 9 weeks of age (data not shown) highlighting the importance of the early postnatal period.

## Discussion

We report two novel findings, first on the interactive effects of prenatal and postnatal maternal depression, and second on the effect of maternal stroking, on *NR3C1* 1-F promoter methylation, in young children. The interaction between prenatal and postnatal depression arose because the association between maternal depression measured at four postnatal time points and *NR3C1* 1-F promoter methylation was stronger in infants who had been exposed to low levels of maternal depression *in utero*. The effect of maternal stroking was seen only in those infants exposed to the combination of low prenatal and high postnatal maternal depression.

Although the sample size of the study was modest, we reduced the risks arising from multiple analyses by examining only one CpG site, prespecified from other studies in the field. Previous studies in humans had also identified maternal depression as a predictor of *NR3C1* 1-F promoter methylation, which we measured prospectively both pre- and postnatally. The measure of maternal stroking was by self-report, and it remains to be established whether observed maternal stroking has the same effect. However, observational measures are generally limited in the studies of human development by restricted coverage over place and time, and we have previously used this measure to show that maternal stroking reverses the effects of prenatal depression on physiological and behavioral reactivity at 29 weeks.^22^ We did not test duplicate DNA samples, so any instability in methylation levels may have contributed unmeasured error to the analyses. The majority of the DNA extracted from whole saliva has been shown to originate from blood leukocytes^33, 34^ and previous studies on NR3C1 methylation have generated similar results by utilizing the DNA from brain^2, 9^ and leukocytes.^10, 14, 16, 17, 18^ These data further support that adversities in early life may both be epigenetically reflected in the central nervous system and in the peripheral tissues (like leukocytes).

To the best of our knowledge, this is the first study in humans or in animals to examine the interactive effects of pre- and postnatal depression on DNA methylation. The findings reported in this paper that the infants of mothers with low prenatal depression were vulnerable to the effects of postnatal depression are consistent with an interplay between prenatal and postnatal environments seen throughout biology. From the effects of exposure to chemical traces of a predator on the offspring of the freshwater crustacean Daphnia, to the long-term effects of restricted fetal growth in humans, prenatal exposure to a risk can confer protection from the effects of postnatal experiences.^35, 36^ In general terms, this is consistent with the fetal origins hypothesis of human disease that proposes that *in utero* environmental exposures lead to modifications in fetal development, which are adaptive where the subsequent postnatal environment is similar. Discontinuities between prenatal and postnatal environments create vulnerability. This effect is best exemplified in the associations of low fetal growth with diabetes and hypertension over several decades, that are thought to arise from fetal adaptations that confer advantage in food-scarce environments but create risk in western food-rich environments.^35^ Low birth weight is also associated with adolescent depression in the presence of childhood adversities, consistent with the hypothesis.^37^ Possible mechanisms for the interplay between prenatal and postnatal influences include differential gene expression of the kind shown at the intron microsatellite in the serotonin transporter.^38^

Building on our previous work on reversal by maternal stroking of behavioral outcomes associated with prenatal depression and anxiety,^22^ we now show a reduction of *NR3C1* gene methylation associated with maternal stroking. These findings support the role of epigenetic mechanisms linking early-life stress with long-term effects,^3^ and highlight the importance of translational research in linking the studies in animals to humans, with considerable implications for our understanding of the earliest origins of neurobiological and behavioral development, and psychiatric disorders. Equally they imply new directions for animal models. In addition to the studies of single pre- or postnatal stressors, the effects of successive stressors need to be examined, in particular, to test for modification by prenatal stress of effects of postnatal stress, and to establish mechanisms. Similarly, not enough is yet known about the ability of postnatal tactile stimulation to reverse the effects of pre- and postnatal stressors, and about associated epigenetic mechanisms. More broadly, human studies, informed by animal models, have the potential to inform the design of animal investigations to bring them closer to the human condition.
